# Determination of Enrofloxacin and Ciprofloxacin Residues in Five Different Kinds of Chicken Tissues by Dispersive Liquid–Liquid Microextraction Coupled with HPLC

**Published:** 2018

**Authors:** Najmeh Rezaee Moghadam, Seyed Rafie Arefhosseini, Afshin Javadi, Farzaneh Lotfipur, Masood Ansarin, Elnaz Tamizi, Mahboob Nemati

**Affiliations:** a *Drug Applied Research Center, Tabriz University of Medical Sciences, Tabriz, Iran.*; b *Department of Biochemistry and Diet therapy, Faculty of Nutrition and Food Sciences, Tabriz University of Medical Sciences, Tabriz, Iran. *; c *Food and Drug Safety Research Center,Health Management and Safety Promotion Research Institute, Tabriz University of Medical Sciences,Tabriz, Iran. *; d *Department of Food Hygine, Faculty of Veterinary Medicine, Tabriz Azad Islamic University, Tabriz, Iran. *; e *Department. of Pharmaceutical and Food Control, Faculty of Pharmacy, Tabriz University of Medical Sciences, Tabriz, Iran.*

**Keywords:** Enrofloxacin, Ciprofloxacin, Dispersive liquid–liquid microextraction (DLLME), HPLC, Chicken

## Abstract

Contamination of food producing animals by veterinary drug residues, particularly quinolones, is an essential issue in food safety that causes increasing concern in consumers. The aim of this study was to investigate the occurrence of enrofloxacin and its main metabolite, ciprofloxacin, in chicken tissue samples slaughtered in Tabriz, Iran. Totally 250 samples including liver, muscle, gizzard, heart, and skin were studied. Dispersive liquid-liquid microextraction technique (DLLME) was used as a simple, high performance, low-cost, and fast sample pre-treatment method followed by a high-performance liquid chromatography with UV detection for quantitative analysis. The residues of enrofloxacin were detected and quantified in 26 liver (52%) and 10 skin (20%) samples and ciprofloxacin residues were detected in 3 skin (6%) samples and accurately determined in 15 liver (30%) samples; however they were not detected in gizzard, heart, and muscle samples. The results showed the accumulation of enrofloxacin and ciprofloxacin residues in chicken liver and skin.

## Introduction

Enrofloxacin(1-cyclosporyl-6-fluoro-1, 4dihydro-4-oxo-7-[4ethyl-1-piperazinyl]-3-quinoline carboxylic acid) is a synthetic fluoroquinolone antimicrobial agent which has a wide spectrum activity against Enterobacteriaceae and other Gram-negative bacteria and some activity against certain Gram positive cocci (1-3). Enrofloxacin is taken orally in chicken, turkeys, pigs, and cattle (with food, milk replacement and/or in drinking water), or administered parenterally through intramuscular injection to pigs or subcutaneous injection to cattle. Ciprofloxacin is the main metabolite of enrofloxacin and it appears in various ratios in foodstuffs after the administration of enrofloxacin (4).

As a result of low ionization and expressive lipophilicity, like other fluoroquinolones, enrofloxacin is spread well and fast through all tissues of the organism (5, 6). Altough the antimicrobial mechanism of enrofloxacin is not compeletly understood, it is confirmed that it is a bactericidal agent that inhibits the function of two enzymes including topoisomerase II and topoisomerase IV. Topoisomerase II (DNA- gyrase) is responsible for DNA replication, which is essential for maintaining spherical twist in DNA (6).

Veterinary drugs are used on a large scale as growth promoters or for the prevention and therapy of infectious diseases in food producing animals such as pigs, calves, poultry, and fish because of their good effectiveness (7-9).

The antimicrobial properties of enrofloxacin show that it has advantages for use in poultry. It is used for the treatment of common poultry infections such as mycoplasmal infection, colibacillosis, and pasteurellosis (10). 

Health problems maight occur as a result of excessive use of veterinary drugs in food producing animals, because most of these substances may have some important toxic effects such as genotoxicity, carcinogenicity, immunotoxicity, or endocrine effects on consumers intaking these substances (11). 

The presence of enrofloxacin resides in foodstuffs may cause allergic reaction in hypersensitive individuals and could lead to the increased pathogen resistance to clinical drugs in humans; therefore, they may got important consequences for public health (7). 

The enrofloxacin residue could enter the food supply and change the ecology of the intestinal flora of consumers. It is also partially metabolized to its main metabolite and ciprofloxacin that affects the human intestinal flora, as well (5). Moreover, fluoroquinolone antimicrobials can cause phototoxic skin reaction in humans (12) and chondrotoxic effects on young animals (13) and tendon rupture (14). Additionally, presence of residual amounts of this antibiotic could cause serious difficulties for food processors in food fermentation control (11). Therefore, to ensure human food safety, different countries and regions set various maximum residue limits (MRLs) on fluoroquinolone residues and some countries have more rigid regulations than others (7).

Because of the complexity of biological solid matrices, sample pretreatment and preconcentration are key points in the determination of antibiotics in different animal body tissues (15). Nowadays, sample preparation methods that generate minimum toxic waste and are more environmentally friendly are introduced. These techniques are dispersive liquid–liquid microextraction (DLLME) (16), supported liquid membrane (17), hollow fiber supported liquid membrane (18), solid phase extraction (SPE) (7), pressurized liquid extraction (19) and microwave-assisted extraction (20). DLLME is a relatively novel microextraction method which is introduced by Rezaee *et al.* in 2006 as a high-performance and powerful preconcentration technique. It is simple, quick and in accordance with the green chemistry (20, 21). 

Different bacteriological, chemical, and immuno-enzymatical methods have been reported for the analysis of fluoriquinolone residues in foodstuffs (4). Chemical methods such as high performance liquid chromatography (HPLC) that are used in several laboratories can simultaneously determine different quinolones, with lower limits of detection (LOD) (21). 

As recently antibiotics and veterinary drugs are widely used in food producing animals that can cause harmful effects on human health, the main purpose of this study was to determine the presence of enrofloxacin and ciprofloxacin residues in chicken samples in Iran using a powerful separation technique, such as HPLC, coupled with a UV detector. In this study, DLLME methodology has been proposed to increase sample preparation throughput.

## Experimental

*Reagents*

Enrofloxacin and ciprofoxacin were purchased from Fluka Biochemica-Sigma–Aldrich (Stein-heim, Germany). Chloroform, phosphoric acid and sodium hydroxide were obtained from Merck Co. (Darmstadt, Germany). Acetonitrile and methanol were of HPLC grade and purchased from Duksan Pure Chemicals Co. (Gyeonggi-do, Korea). 

*Chicken Samples*

Totally 250 liver, muscle, gizzard, heart, and skin samples were collected from different abattoirs in Tabriz, Iran. Organic chickens were used as blank samples and these samples were analyzed to ensure that they are free of enrofloxacin and ciprofloxacin. The chicken samples were preserved at -20 ºC for further analysis.

*Enrofloxacin and Ciprofoxacin – Added Materials*

Individual stock solutions of enrofloxacin and ciprofloxacin were prepared at a concentration of 100 µg/mL *via* dissolving the accurately weighed amount of each antibiotic in acetonitrile and working standard solutions with a concentration of 10 µg/mL were prepared through diluting the appropriate amount of stock solution with distilled water. Standard solutions were covered with aluminum foil and kept at 4 °C. All spiked solutions were kept at room temperature for 1 h before analysis.

*Apparatus*

The analyses were performed using a KNAUER high performance liquid chromatographic system consisting of a degasser (Biotech model 2003,Onsala, Sweden), an isocratic pump (K-1000, Knauer, Berlin, Germany) and an UV–Vis detector (Knauer K-2005, Berlin, Germany). A perfectsil target-C18 column (4.6 mm × 250 mm, 5µm) was used for separation. The column temperature was set at 25 ºC and the injection volume was 20 µL; the mobile phase made up of acetonitrile and phosphoric acid buffer (0.01 M, pH 3) (25:75% v/v) were utilized with a flow rate of 1.0 mL/min and detection was carried out at 278 nm. A centrifuge (Pheonix, Germany), a vortex mixer (Heidolph, UK), a pH meter (Metrohm, Switzerland) and an oil–less piston vacuum pump (Kawake Airvac, Taiwan) were used as well.

*Sample preparation*

Chicken liver, muscle, gizzard, heart, and skin samples were prepared using a technique previously described by Moema *et al.* (7), with minor modifications. The samples were crushed using a kitchen blender separately and 5 g of each homogenized sample was weighted accurately and transeferd into a 10 mL centrifuge tube. Then, 5 mL of 25 mM phosphoric acid:acetonitrile (30:70 v/v) solution was added to the sample and shaken for 30 s. The samples were centrifuged for 10 min at 4500 rpm at room temperature. The supernatant was filtered through a 0.45 µm membrane filter and transferred into a test tube. In skin samples the accumulated fat content at the top of the test tube was separated first and then the sample was filtered. The pH value of acetonitrile extract was adjusted to 7.0 using NaOH 0.1 N, to obtain the highest extraction efficiencies. 1 mL of the acetonitrile extract was used for DLLME procedure.

*DLLME procedure*

According to the previously reported work by Moema *et al. *(7), the DLLME-HPLC-UV method was done as the following: 5 mL of double distillied water was transferred into a screw-cap glass test tube with a conical bottom. After that, 1.0 mL of disperser solvent (the acetonitrile extract ) was added and 200 µL of extracting solvent (chloroform) was injected rapidly into the mixture. The ternary component solvent system was mixed immediately by vortex mixer for 30 s. Then, the resulted cloudy solution was centrifuged at 4500 rpm for 5 min and the sedimented phase, laden with enrofloxacin and ciprofloxacin, was transferred into the microtube and dried at 25 °C under a gentle stream of nitrogen gas. Finally, the residue was redissolved in 100 µL mobile phase and injected into the HPLC system.

*Method validation*

Since, the previously reported method (7) has been utilized for the analysis of samples, according to the FDA guidelines on the validation of bioanalytical methods (22), the method was partially validated in terms of linearity, accuracy, repeatability, limit of detection (LOD), and limit of quantification (LOQ). 

In order to evaluate linearity of the method for different samples, seven point matrix matched calibration curves were obtained through spiking different blank samples with enrofloxacin and ciprofloxacin in the concentration range of 5 to 500 µg/kg. To illustrate, a spiked sample with a concentration of 30 µg/kg was prepared by adding 15 µL of working standard solutions to 5 g of blank tissue samples; other concentrations were also prepared according to this method. LOD and LOQ of the method for each sample were calculated using the below equations:

*LOD = 3.3× SD/s*

*LOQ = 10 × SD/s*

Where *s* and *SD* were the slope and standard deviation of the y-itercept of three individual calibration curves (23). 

Accuracy and precision studies were carried out using spiked samples with concentrations at the lower, middle, and upper levels of the linearity range. Each spiked sample was analyzed using the method in triplicate and the experimentally derived concentrations were calculated using the obtained peak areas and calibration equation. The accuracy and repeatability of the method were expressed as the percentage of the experimentally derived concentration to the nominal concentration and relative standard deviation (RSD%) of the calculated concentrations, respectively. 

Recovery calculatios were done using the spiked samples with the concenterations covering the linear range. The obtained recoveries were reported as the percentage of the recovered concentration using the DLLME-HPLC method to the known concentration which was added to spike the blank samples. 

And finally, in order to evaluate the suitability of the HPLC method for the sumultanous analysis of enrofloxacine and ciprofloxacine, system suitability parameters including resolution between the peaks, capacity factor, tailing factor, and number of theoretical plates were calculated using the standard smples.

## Result and Discussion

*Method validation*

The linearity parameters of the proposed DLLME-HPLC method have been reported in Table 1. As indicated in this table, the method could detect and quantify the little amounts of enrofloxacin and ciprofloxacin in chicken samples, especially in liver and skin. Since the achieved LOQs were lower than MRL value established by European Union Commission Regulation No 37/2010 – (EU 37/2010), the method could be utilized to inspect these antibiotics› amounts in real samples.

**Table 1:** Analytical performance parameters for determination of enrofloxacin and ciprofloxacin in chicken liver

**LOQ (μg/Kg)**	**LOD (μg/Kg)**	**Correlation coefficient (r)**	**Calibration equation**	**Data point**	**Linear range (μg/Kg)**	**Sample**
9.7	3.2	0.9976	y = 994.94x– 1892.7	7	15 - 100	CIP a - Liver
13.8	4.5	0.9997	y = 823.12x– 2975.5	7	15 - 300	ENR b - Liver
22.1	7.3	0.9989	y = 1363.1 x + 16160	7	30 - 500	CIP - Skin
16.1	5.3	0.9997	y = 811.88 x + 2764.8	7	30 - 500	ENR - Skin

a CIP: ciprofloxacin

b ENR: enrofloxacin

According to the reporetd results in Table 2, the method was accurate and precise enough for the quantification of enrofloxacin and ciprofloxacin in liver and skin samples covering a wide concentration range around the acceptable MRL value. The obtained recoveries for spiked liver samples with concentrations in the linear range were in the range of 93.7 ± 1.05 to 103.7 ± 0.4 % for enrofloxacin and 98.3 ± 0.6 to 101.7 ± 0.9 % for ciprofloxacin and the recoveries of spiked skin samples were between 80.5 ± 1.2 % and 111.0 ± 7.1 % for enrofloxacin and between 82.2 ± 9.9 % and 111.8 ± 4.9 % for ciprofloxacin. 

**Table 2:** The accuracy and precision of the DLLME-HPLC method for the analysis of enrofloxacin and ciprofloxacin in chicken liver and skin samples (n = 3)

**Repeatability (RSD%)**	**Accuracy (%)**	**Concentration (μg/Kg)**	**Sample**
8.8	96.6±1.3	15	CIP a - Liver
1.2	100.3±1.3	45	
7.4	99.9±7.4	100	
8.6	92.2±8.0	15	ENR b - Liver
2.6	95.1±2.6	100	
3.5	99.8±3.4	300	
11.3	87.9±9.9	30	CIP - Skin
2.4	101.2±2.4	180	
0.4	99.7±0.4	500	
17.0	89.2±15.2	30	ENR - Skin
2.3	101.8±2.3	180	
1.3	100.9±1.3	500	

a CIP: ciprofloxacin

b ENR: enrofloxacin

Besides, as it can be seen in Table 3, the applied HPLC method was suitable for the intended propose owing to the capacity factor of more than 1, tailing factor of less than 2 and reolution factor of more than 1.5.

**Table 3:** System suitability parameters of the proposed method for the quantification of enrofloxacin and ciprofloxacin

**Analyte**	**Capacity factor**	**Tailing factor**	**Resolution factor**	**Theoretical plates number**
Enrofloxacin (Retention time = 14.3 ± 0.4)	4.1	1.7	2.5	4900
Ciprofloxacin (Retention time = 10.9 ± 0.2)	3.0	1.3		4233

It shoud be mentioned that all the blank samples were previously analyzed to ensure the absence of quinolone residues; also it is worth mentioning that since the method could not detect the analytes in the real gizzard, heart and muscle samples and consequently were not applied to quantify them in mentioned samples, the method validation results in these matrices were not completely reported; however, the relative recoveries of enrofloxacin and ciprofloxacin were 84.0 ± 7.1 %, 83.0 ± 6.2 % in gizzard samples, 93.0 ± 6.1 %, 90.0 ± 9.9 % in heart samples and 97.0 ± 5.6 %, 94.0 ± 5.5 % in muscle samples. 

*Real sample analysis*

**Figure 1:**
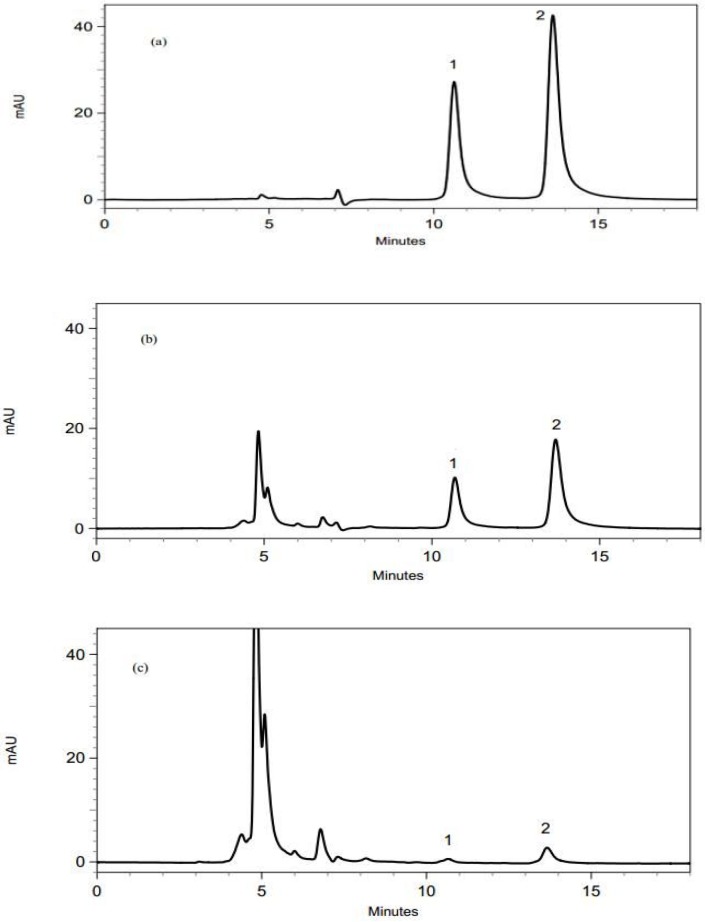
The chromatograms obtained using the proposed DLLME-HPLC method; (a) a standard solution with a concentration of 1 µg/mL, (b) a spiked liver sample with enrofloxain and ciprofloxacin at a concentration of 0.5 µg/mL, (c) a real liver sample containig enrofloxain and ciprofloxacin. Peak identification: (1) ciprofloxacin, (2) enrofloxacin

The validated method was applied to determine the enrofloxacin and ciprofloxacin residues in 50 liver, 50 heart, 50 muscle, 50 gizzard and 50 skin samples. Figure 1 shows the chromatograms of liver samples. It should be mentioned that since there is not any significant differences among the obtained chroromatograms from the analysis of different samples, only chromatogram of liver samples were brought.

**Table 4:** The results for determination of enrofloxacin and ciprofloxacin in real chicken tissue samples

**Enrofloxacin**			**Ciprofloxacin**		
**Tissue**	**Mean(µg /kg)**	**Range**	**Tissue**	**Mean(µg /kg)**	**Range**
Liver	131.6 ± 89.5	16.7 - 296.7	Liver	24.8 ± 23.5	9.8 - 93.3
Skin	21.7 ± 5.3	16.2 - 30.7	Skin	NQ a	―
Muscle	ND	―	muscle	ND b	―
Heart	ND	―	Heart	ND*	―
gizzard	ND	―	gizzard	ND*	―

a NQ: Detected but not quantified (< LOQ)

b ND: Not detected

According to the reported results in Table 4, enrofloxacin residues were detected and quantified in 26 liver (52%) and 10 skin (20%) samples and ciprofloxacin residues were determined in 15 liver (30%) samples. However, they were not detected in gizzard, muscle, and heart samples. It is worth saying that the method detected ciprofloxacin residues in 3 skin (6%) samples, but since their amounts were less than calculated LOQ values, their exact concentrations could not be reported.

The Iranian National Standards Organization has not fixed MRL value for enrofloxacin in food samples. According to the EU 37/2010 document, the MRL was established 200 µg/kg for liver, 100 µg/kg for muscle and 100 µg/kg for skin and fat (sum of enrofloxacin and ciprofloxacin) (24). The sum of enrofloxacin and ciprofloxacin residues were above than the MRL established by European Union in 28% of liver samples; however, the drug residues were below the MRL in skin samples. Therefore, the obtained results attract some attention to the fact that it is necessary to give scientific information to poultry breeders about the withdrawel period, a duration from the time antibiotic administered until it is legal to slaughter the animal. Also it seems that organizations which are responsible for the food quality control must do more serious supervisions and inspections. 

There are some reported studies about determination of enrofloxacin and ciprofloxacin in animal tissue samples as shown in Table 5. A closer look at the table indicates that the applied DLLME-HPLC method represented lower LOD amounts in comparison to the some other studies which used SPE as a preconcentration technique (25, 26). Additionally, the utilized method resulted in lower LOD values compared to the previous works applied DLLME in swine muscle (LOD of 16.4 µg/kg for enrofloxacin) (20) and chicken liver (LODs of 5 and 16 µg/kg for enrofoxacin and ciprofloxacin, respectively) (7). Therefore, it can be said that the present method could be utilized to detect lower amounts of enrofoxacin and ciprofloxacin in chicken tissues, especially in liver and skin.

**Table 5:** Comparision among the differnt analytical methods utilized to determine enrofloxacin and ciprofloxacin in solid samples

**Method**	**Matrix**	**Analyte**	**LOD** **(µg/kg)**	**Regression coefficient**	**Recovery (%)**	**Reference**
SPE-HPLC-UV^a^	Chicken muscle	Enrofloxacin	5	0.9918	99.1 – 99.8	(27)
		Ciprofloxacin	8	0.9982	99.2 – 100.3	
SPE-CE-MS^b^	Chicken muscle	Enrofloxacin	18	0.9996	65.0	(28)
		Ciprofloxacin	―	―	―	
SPE-LC-UV^c^	Chicken tissue	Enrofloxacin	5	0.9984	85.0	(25)
		Ciprofloxacin	5	0.9986	70.0	
SPE-LC-MS	Chicken tissue	Enrofloxacin	0.2	0.9995	85.0	(25)
		Ciprofloxacin	0.5	0.9992	70.0	
SPE-CE-DAD^d^	Chicken	Enrofloxacin	10	0.9999	74.0	(29)
		Ciprofloxacin	25	0.9997	54.0	
DLLME-LC-DAD^e^	Swine muscle	Enrofloxacin	16.4	0.9992	96.1 – 101.7	(20)
		Ciprofloxacin	―	―	―	
DLLME-LC-DAD	Chicken liver	Enrofloxacin	5	0.9959	98.0 – 100.0	(7)
		Ciprofloxacin	16	0.9962	89.0 – 96.0	
DLLME-HPLC-UV	Chicken liver	Enrofloxacin	4.5	0.9997	93.7 – 103.7	This study
		Ciprofloxacin	3.2	0.9976	98.3 – 101.7	
DLLME-HPLC-UV	Chicken skin	Enrofloxacin	5.3	0.9989	80.5 – 111.0	This study
		Ciprofloxacin	7.3	0.9997	82.2 – 111.9	

a Solid-phase extraction- high-performance liquid chromatography- ultraviolet.

b Solid-phase extraction- capillary electrophoresis- mass spectrometry.

c Solid-phase extraction- liquid chromatography- ultraviolet.

d Solid-phase extraction- capillary electrophoresis- diode array detection.

e Dispersive Liquid–liquid microextraction- liquid chromatography- diode array de

## Conclusion

In the present study, a DLLME-HPLC method was successfully applied for the extraction and quantification of enrofloxacin and ciprofloxacin in different tissues of chicken intended for human consumption. This was a simple and sensitive method with an adequate linearity, accuracy, precision, and recovery that led to the significant reduction in organic solvent consumption. All these advantages make the proposed method as an appropriate technique applicable in the wide range of routine analytical laboratories. Using this method enrofloxacin and ciprofloxacin were found in some chicken tissues which raises the awareness about the need to consider stricter legislation on the use of veterinary drugs in poultry in Iran.
